# Progress in Surgery

**Published:** 1898-03-12

**Authors:** 


### Progress in Surgery.

### SURGERY OF THE INTESTINES.

**Statistics of Intestinal Operations** are given by Wölfler.[1] The mortality of resection of the intestines in general has been 39·5 per cent. The results of the last seven years show only 6 per cent. of improvement. Out of 221 resections, 84 on the small intestines are attended with a mortality of 30 per cent.; 69 of ileo-cæcal portion with 42 per cent.; and 81 of the large intestine with 49 per cent.; the prospect of an operative success diminishing with the decreasing length of the mesentery. Mikulicz insists that the nature of the condition bears an important relation to the operative result, and this is borne out by Wölfler. Thus 78 resections of the intestine for artificial anus gave 78 per cent. of cure; there were 73 per cent. cures out of 34 cases of tuberculosis of the intestine; cicatricial contractions gave 65 per cent. of cures out of 20 cases; and new growths of the intestine had a percentage of only 46 cures out of 114 cases, the majority being carcinoma. The operative results of resection of the pylorus have much improved of late years. Mikulicz, Krönlein, and Kocher have lately performed the operation in nine to ten cases without a mishap. According to the statistics obtained by the author from 15 well-known operators, the mortality of all their resections of the pylorus, 92 cases, is 56·4 per cent.; from 1888 to 1896, in 173 operations, only 31·2 per cent. Resection of the pylorus for cicatricial contraction gave a mortality of about 10 per cent. The existence of 24 patients has been prolonged after operation from two to eight years. Forty-five cases of gastro-enterostomy prior to 1888 gave a mortality of 55·6 per cent. (56·4 in pylorectomy). After 1888 the mortality fell for gastro-enterostomy, 219 operations, to 36 per cent. (in pylorectomy 31·2 per cent.) According to Zeller, the mortality sank from 70 per cent. (1881 to 1885) down to 38·7 per cent. (1886 to 1892). Wölfler has knowledge of 15 cases of gastro-enterostomy in which life was prolonged more than one year; the longest time was two and a quarter years. Pyloro- and entero-plasty have been attended with a mortality of 26 per cent. out of 50 cases in the author's statistics; 26 per cent. out of 51 cases according to Haberkaut. The mortality of gastrostomy from 1849 to 1883 was 77·7 per cent.; from 1883 to 1886, 37 per cent.

**Mesenteric Cysts** are described by Moyniham,[2] who gives many references to cases, and divides them as follows: (1) Serous cysts. (2) Chyle cysts. (3) Hydatid cysts. (4) Blood cysts. (5) Dermoid cysts. (6) Cystic malignant disease—cystic sarcoma. In addition to these true cysts are certain pathological conditions of the lymphatics of the mesentery, which have been mentioned by Angagneur and others. These are: (1) Lymphatic varices. (2) Lymphatic nævus. (3) Cavernous lymphangioma. Serous cyst may be multilocular or unilocular, and the origin may be two-fold: (1) From hæmorrhage between the layers of the mesentery. (2) From a dilatation of a portion of a lymphatic vessel, occluded as the result, probably, of some inflammatory disturbance. Chyle cysts are formed: (1) By a dilatation of the lacteals of the mesentery—an exaggerated condition of lymphatic varix. (2) In degenerated glands—as asserted by Rokitansky and Virchow. (3) By a dilatation of the receptaculum chyli. Chyle cysts may be either unilocular or multilocular. The origin of serous cysts of the mesentery from hæmorrhage has been insisted upon by Ricket. A hæmorrhage into the mesentery, then, may result in: (1) A pure blood cyst. (2) A sero-sanguineous cyst—serous cyst. (3) A hæmorrhage into a pre-existing cyst. (4) A hæmorrhage into a mesenteric growth. (5) A solid tumour. The most characteristic signs of a mesenteric cyst are: (1) Prominence of the fluctuating tumour towards the umbilicus. (2) Great mobility, especially in the transverse direction, and the possibility of rotation round a central axis. (3) The presence of a zone of resonance around and a belt of resonance across the cyst. Many of these cysts remain undiscovered until operation for some more serious condition, but clinically they may be divided into: (1) Chronic cases, where complaint is made chiefly of an abdominal tumour, but with constipation and perhaps vomiting, and pain more or less acute, according to the rapidity of growth of the tumour. (2) Acute cases, in which the onset of acute intestinal obstruction first draws the attention of the patient and practitioner to the abdomen. The causes which have been suggested as bringing about acute obstruction in these cases are: (1) Irritation of the solar plexus by pedicle of cyst. (2) Compression of the intestine, amounting in some cases to gangrene. (3) Formation of adhesions, and strangulation by them. (4) Onset of acute peritonitis, owing to rupture of the cyst or its pedicle. (5) Strangulation over a band formed by the pedicle of the cyst. These cysts are most likely to be confounded with ovarian or peri-ovarian cysts; tumours of the kidney, liver, spleen, &c.; cysts of the urachus, pancreatic cysts, and lipomata of the mesentery; but attention to the signs above given will generally succeed in establishing a diagnosis. Cases not operated upon may terminate: (1) The cyst, especially when hydatid, may retrogress, or remain quiescent. (2) Perforation may occur into the intestine, and the contents of the cyst be discharged. (3) Perforation of the cyst or pedicle into the peritoneum, and the onset of acute peritonitis. (4) Extreme emaciation may end in death, the result most probably of compression of, or other interference with, the lacteals. (5) Acute intestinal obstruction may ensue. Operative treatment is necessary in both acute and chronic cases. As soon as the abdomen is opened two methods of procedure may be adopted: 1. Stitch the cyst wall to the parietal peritoneum and open and drain the cavity—generally best in the acute cases. 2. The cyst may be removed after aspiration, either by cutting through the opening layers of peritoneum in the pedicle, inverting the edges and applying a series of Lembert sutures, draining the peritoneum or not, according as may seem fit; or, if no pedicle is found, the peritoneum may be incised over the centre of the tumour and the cyst enucleated—these procedures being generally best for chronic cases.

**Solid Mesenteric Tumours**, according to Harris and Hertzog,[3] are generally carcinomatous. Of 57 cases reported they find the histological type as follows: Carcinomata, 16 cases; lipomata, 10 cases; sarcomata, 7 cases; myxo-lipomata, 3 cases; fibromata, 2 cases; fibro-cartilaginous tumours, 2 cases; lipoma with calcareous masses, fibro-lipoma, fibroma with calcareous degeneration, fibro-myxoma partially ossified, osseous tumour, chylangioma, adeno-lymphoma, malignant lymphoma, fibro-sarcoma, lympho-sarcoma with colloid degeneration, one case each; and no data or indefinite, seven cases. In the majority of cases reported treatment has been symptomatic or palliative (34 cases), and death invariably occurred. Paracentesis abdominis was made four times, and a radical operation, laparotomy with removal of the tumour, performed 18 times. Once an exploratory laparotomy was made, followed speedily by death from septicæmia. As regards final results: Death was reported in 40 cases, no data given in 7 cases, and recovery after operation in 10 cases. Of these last 10 cases only 3 were malignant tumours.

**Intestinal Obstruction.** — M'Ardle[4] formulates the following rules for operation in this condition: (1) Follow engorged coil of intestines upwards and downwards until point of obstruction is reached, or turn out all the intestines. (2) Remove all fluid from Douglas' pouch and loins by irrigation with sterile water. (3) Restore colour of bowel, and establish peristaltic movements by heating with neutral saline solution. The removal of the primary cause of intestinal obstruction is not always followed by relief of symptoms. (4) Should there be difficulty in returning intestines, elevate pelvis in Trendelenburg's position, or, if necessary, open and wash out. (5) The most important rule of all is—when called to a case of complete obstruction of the bowel, with evidence of peritoneal effusion, operation should be performed at once. Stokes[5] refers to two cases of ante-operative asphyxia following acute intestinal obstruction. Both patients were in the horizontal position while they were being anæsthetised, which allowed the stomach to act as a reservoir for the intestinal fluid. Though the position of the patients was changed the moment the fluid commenced to ooze up, it proved totally ineffectual. In one case the patient was drawn over the end of the table and turned well over on her side; and, in the second case, the patient was literally hung up by his feet. In both cases artificial respiration was kept up for about thirty minutes, and during this time the fluid poured from the mouth and nose in such a torrent that it was impossible to keep it from running back into the trachea. The author therefore maintains that in such cases the patient should be well propped up and kept in this position, not only during the administration of the anæsthetic and the operation, but especially until the distension of the intestines is reduced. The Trendelenburg posture, or even a horizontal position, is dangerous, because each allows of the gravitation of the intestinal contents towards the mouth. Kammerer[6] has added ten cases of his own, of obstruction produced by Meckel's diverticulum, to the statistics of Neumann and Boldt, and finds that of 66 cases the diverticulum had an attachment to the mesentery in 33 cases, and there were 13 attachments to the umbilicus, 8 to the abdominal wall, and 12 to the intestinal tract and other abdominal viscera. Page[7] relates a case of obstruction treated by Paul's tube, and thinks this method useful where there is much distension and the condition of the patient does not admit of prolonged operation.

[1] Annals of Surg., May, 1897. [2] Ibidem, July, 1897, pp. 1-30. [3] Ibidem, pp. 66-82. [4] Dublin Journ. Med. Sci., Oct. 1, 1897, p. 273. [5] Annals of Surg., Sept., 1897, p. 340. [6] Ibidem, Aug., 1897, p. 179. [7] Brit. Med Jour., May 29, 1897.

